# Nutraceutical Strategies for Suppressing NLRP3 Inflammasome Activation: Pertinence to the Management of COVID-19 and Beyond

**DOI:** 10.3390/nu13010047

**Published:** 2020-12-25

**Authors:** Mark F. McCarty, Simon Bernard Iloki Assanga, Lidianys Lewis Luján, James H. O’Keefe, James J. DiNicolantonio

**Affiliations:** 1 Catalytic Longevity Foundation, San Diego, CA 92109, USA; markfmccarty@gmail.com; 2 Department of Research and Postgraduate in Food, University of Sonora, Centro 83000, Mexico; ilokiassanga@gmail.com (S.B.I.A.); lidianys1@yahoo.es (L.L.L.); 3 Mid America Heart Institute, Kansas City, MO 64111, USA; jokeefe@saint-lukes.org

**Keywords:** inflammasomes, NLRP3, phycocyanobilin, lipoic acid, ferulic acid, N-acetylcysteine, berberine, glucosamine, zinc, macular degeneration, COVID-19

## Abstract

Inflammasomes are intracellular protein complexes that form in response to a variety of stress signals and that serve to catalyze the proteolytic conversion of pro-interleukin-1β and pro-interleukin-18 to active interleukin-1β and interleukin-18, central mediators of the inflammatory response; inflammasomes can also promote a type of cell death known as pyroptosis. The NLRP3 inflammasome has received the most study and plays an important pathogenic role in a vast range of pathologies associated with inflammation—including atherosclerosis, myocardial infarction, the complications of diabetes, neurological and autoimmune disorders, dry macular degeneration, gout, and the cytokine storm phase of COVID-19. A consideration of the molecular biology underlying inflammasome priming and activation enables the prediction that a range of nutraceuticals may have clinical potential for suppressing inflammasome activity—antioxidants including phycocyanobilin, phase 2 inducers, melatonin, and N-acetylcysteine, the AMPK activator berberine, glucosamine, zinc, and various nutraceuticals that support generation of hydrogen sulfide. Complex nutraceuticals or functional foods featuring a number of these agents may find utility in the prevention and control of a wide range of medical disorders.

## 1. Mechanisms Involved in the Priming and Activation of Inflammasomes

Inflammasomes are protein complexes that assemble in response to certain pro-inflammatory signals and induce the self-activation of caspase-1; this protease, in turn, cleaves pro-interleukin-1β and pro-interleukin-18 to generate the active forms of these mediators, which exert a crucial pro-inflammatory and pro-apoptotic effect in many pathologies [1,2]. Inflammasomes can also induce a type of cell death known as pyroptosis; this results from caspase-1-mediated cleavage of the protein Gasdermin-D, which subsequently forms a pore in the plasma membrane that enables extracellular efflux of cytokines such as interleukin-1β, and that also can also induce cell swelling and death [3]. A range of different types of inflammasomes have been characterized; these include the NLR-subset (NLRP1, NLRP3, NAIP/NLRC4), as well as the AIM2 and IFI16 inflammasomes [4]. This review focuses on the NLRP3 inflammasome, which has received by far the most study.

Interleukin-1β plays a mediating role in atherosclerosis, myocardial infarction, and heart failure; a phase III study of the monoclonal antibody canakinumab that targets this cytokine found that, in patients who had previously experienced a myocardial infarct and had elevated plasma C-reactive protein, canakinumab administration significantly lowers risk for a subsequent Myocardial infarction (MI) [5,6]. With respect to interleukin-18, evidence suggests that this is a crucial driver of the retinal pigment cell apoptosis responsible for the untreatable dry form of age-related macular degeneration [7,8,9]. Inflammasomes are suspected to play a pathogenic role in the complications of diabetes, in a host of neurodegenerative disorders, in autoimmune disorders including rheumatoid arthritis, and in periodontal disease [10,11,12,13,14,15,16,17,18,19]. Inflammasomes contribute to the pathogenesis of chronic skin disorders such as psoriasis and acne [20,21,22,23]. They have also been linked to asthma and allergic inflammation [24,25,26,27]. And inflammasomes are mediators of certain acute inflammatory conditions, such as gout and the respiratory distress syndrome associated with the late stage of certain viral lung infections, including COVID-19 [28,29,30,31,32,33,34,35,36,37,38]. Hence, feasible measures that could prevent inflammasome activation–most particularly, safe nutraceutical measures–might be of considerable value in preventive and therapeutic medicine.

The NLRP3 inflammasome consists of the protein NLRP3 complexed with the proteins ASC and caspase-1, along with several accessory proteins, including NEK7 [39]. Formation of NLRP3 inflammasomes typically requires a priming step, in which the activated transcription factor Nuclear factor kappa beta (NF-kappaB) drives increased expression of NLRP3 and of pro-interleukin-1β and -18. Subsequent activation of NLRP3 inflammasomes, in which the NLRP3/ASC/caspase-1 complex assembles, is typically triggered by oxidative stress, a drop in intracellular potassium, and/or a disruption of lysosomes, which release cathepsins [40,41].

The role of oxidative stress in triggering NLRP3 inflammasome assembly is well characterized. This assembly requires an interaction between thioredoxin interacting protein (TXNIP) with NLRP3 [42,43]. However, much of the cellular pool of TXNIP is tied up in covalent complexes with thioredoxin. When thioredoxin is in its usual reduced configuration, its C32 can nucleophilically attack a disulfide in TXNIP, forming a disulfide bond linking thioredoxin with TXNIP; in this configuration, TXNIP is incapable of interacting with NLRP3 [44,45]. However, when cellular oxidant production accelerates, this disulfide bond is broken as thioredoxin assumes its oxidized configuration with a C32–C35 disulfide. Indeed, the cellular pool of thioredoxin is converted to this configuration as thioredoxin is employed to reduce other oxidized cellular proteins. Reconverting thioredoxin to its reduced form is the task of thioredoxin reductase, which employs NADPH as a reductant [46]. Hence, oxidative stress tends to free up TXNIP so that it can interact with NLRP3, whereas efficient thioredoxin reductase activity helps to maintain thioredoxin in a reduced state so that it can bind TXNIP, thereby preventing inflammasome activation.

The role of plummeting intracellular potassium in the assembly of NLRP3 inflammasomes is still poorly understood at the molecular level, but the phenomenon is well established [1,47]. Potassium depletion is necessary for the kinase NEK7 to bind to NLRP3, an essential step in inflammasome formation [48]. A key mediator of cellular potassium depletion in many circumstances is the P2X7 receptor (P2X7R), whose natural agonist is extracellular ATP. During inflammation and cell damage, ATP is often released to the extracellular space, where it can interact with P2X7R. The activation of this receptor leads to the formation of a pore in the plasma membrane, which allows potassium to stream out, and calcium to stream in [49,50,51]. Activated P2X7R not only promotes the potassium depletion required for inflammasome formation, but also can stimulate generation of oxidative stress via NADPH oxidase activation and it has an adverse impact on mitochondrial function [52,53,54,55].

The role of lysosomal destabilization in inflammasome formation is likewise only sketchily understood. Exposure of cells to certain pathogenic crystals or particulates—including monosodium urate crystals and silica—can induce low-level permeabilization of lysosomes that somehow triggers potassium efflux [56]. In addition, extra-lysosomal cathepsin B physically interacts with NLPR3 on the endoplasmic reticulum membrane, prior to the association of NLPR3 with ASC; this interaction may be of mechanistic importance, as knockout of cathepsin B impairs inflammasome activation induced by a range of mediators [57].

This concise sketch of inflammasome activation enables us to deduce that certain measures would tend to block inflammasome formation—among them: inhibition of NF-kappaB activation; up-regulated expression of thioredoxin and of thioredoxin reductase; down-regulated expression of TXNIP; inhibition of the generation of superoxide, most particularly that produced by NADPH oxidase complexes; and boosting the expression of enzymes that catabolize hydrogen peroxide. Fortuitously, certain nutraceuticals have the potential to accomplish these aims.

## 2. NLRP3 Inflammasome Suppression via Phase 2 Induction

Phase 2 inducers are agents that trigger the increased expression of a wide range of antioxidant, detoxifying, and cytoprotective enzymes [58]. Many of them accomplish this by interacting covalently with cysteine groups of Keap1, a protein that binds to the transcription factor nrf2, retaining it in the cytoplasm and promoting its proteasomal degradation [59,60]. When phase 2 inducers—or their electrophilic metabolites—bind to Keap1, nrf2 is freed up to migrate to the nucleus and promote the transcription of many cytoprotective enzymes, the promoters of whose genes contain antioxidant response elements capable of binding nrf2. Of key importance to our discussion is the fact that phase 2 induction boosts the expression of both thioredoxin and thioredoxin reductase, as well as that of glutathione peroxidase, capable of eliminating hydrogen peroxide [61,62,63,64,65]. Nutraceutical phase 2 inducers that have shown important clinical utility include lipoic acid, ferulic acid, melatonin, and sulforaphane [66,67,68,69,70,71,72,73,74].

Whereas ferulic acid clearly stimulates nrf2-driven transcription, it is not clear that it interacts with Keap1; hence, its activity may be complementary to that of phase 2 inducers that do bind Keap1. Ferulic acid is of particular interest in light of the fact that, in addition to its phase 2 activity, it possesses a still poorly understood anti-inflammatory action that in many pro-inflammatory circumstances tends to suppress NF-kappaB activity [75]. In other words, it has potential for influencing both the NF-kappaB-dependent priming phase of inflammasome formation, as well as the activation phase. Ferulic acid’s anti-inflammatory effect is still imperfectly understood, but may reflect reduced expression and/or diminished activity of the coupling factor MyD88, a key mediator in many pro-inflammatory signaling pathways that activate NF-kappaB and the stress-induced MAP kinases [75,76,77]. Not surprisingly, ferulic acid has been found to suppress NLRP3-dependent inflammasome formation in various cellular models and in rodents [78,79,80,81,82,83]. Ferulic acid has good oral bioavailability and, as with sodium ferulate, has long been used in cardiovascular medicine in China [84]. Whereas its properties have been broadly explored in pre-clinical studies, its impacts as a nutraceutical have received minimal study to date. However, one recent controlled clinical study found that oral administration of ferulic acid (500 mg twice daily) decreased serum C-reactive protein by about a third in hyperlipidemic subjects-evidently indicative of significant systemic anti-inflammatory potential [85]. Ferulic acid, often in conjugated forms, is one of the most widely distributed of phytochemicals in foods; gut bacteria can convert anthocyanins to this compound, and ferulic acid may be the primary mediator of the health benefits associated with anthocyanin ingestion—as intact anthocyanins are barely absorbed [75].

The tryptophan-derived hormone melatonin—often viewed as a nutraceutical insomuch as it can be administered orally and is sold in capsule form over-the-counter—has phase 2 activity that appears to reflect up-regulation of nrf2 at the transcriptional level [86,87,88]. This may reflect the fact that the promoter of the nrf2 gene contains E boxes that bind the “clock” transcription factor Bmal1, in conjunction with the protein Clock, and this drives nrf2 transcription [89,90]. The expression of Bmal1 and other so-called clock genes varies with a diurnal rhythm in many types of cells; a nocturnal surge of melatonin produced by the pineal gland somehow functions to coordinate this diurnal variation with light/dark cycles [91,92]. Bmal1 is the key driver of the diurnal cycles of clock genes, and melatonin appears to boost the amplitude of its cyclic expression, possibly by enhancing the activity of the RORα transcription factor that drives its transcription [93,94]. Studies have found that melatonin loses its protective antioxidant/anti-inflammatory properties when RORα expression is blocked [95,96,97]. Nonetheless, the initial notion that melatonin acts as a direct agonist for RORα has been disproved. The molecular biology underlying melatonin’s up-regulatory impact on RORα activity remains obscure, though this presumably reflects signaling transmitted by the melatonin membrane receptors MT1 and MT2, which are seven-pass receptors coupled to heterotrimeric G proteins [94,98]. One intriguing study has provided evidence that melatonin boosts the nuclear importation of RORα, which would be expected to amplify its activity as a transcription factor [95]. The observation that melatonin concurrently boosts RORα mRNA could be explained by the fact that RORα promotes transcription of the gene encoding Bmal1, which in turn drives transcription of the RORα gene.

Like ferulic acid, melatonin has an effect independent of phase 2 induction that suppresses NF-kappa activation in many circumstances [99,100,101]. This may reflect the fact that Bmal1, whose expression melatonin boosts, binds to the promoter of the sirt1 gene and drives its transcription; sirt1 activity opposes NF-kappaB activity by de-acetylating p65 [95,102,103]. In combination with phase 2 induction, this makes melatonin particularly promising as an agent for down-regulating inflammasome activity, opposing both priming and activation steps. Indeed, a great many studies in cells cultures or rodents have shown that melatonin can suppress the activation of NLRP3 inflammasomes [104,105,106].

Several phase 2-inducible enzymes required for disposing of hydrogen peroxide and maintaining thioredoxin in a reduced state—including glutathione peroxidase and thioredoxin reductase—are selenium-dependent [107]. Hence, assuring adequate selenium status may help prevent inflammasome overactivity in regions where soil selenium and dietary selenium intakes are low. Selenium status has been shown to modulate NLRP3-dependent inflammasome activity in mice [108].

## 3. Controlling Oxidative Stress with Phycocyanobilin

The superoxide production that triggers inflammasome activation is often attributable in large part to NADPH oxidase complexes [109,110,111,112,113,114,115,116,117,118]. Moreover, oxidants generated by these complexes often play a co-factor role in signaling pathways that activate NF-kappaB, possibly by expediting the assembly of certain protein signaling complexes [119,120]. The enzyme heme oxygenase-1 (HO-1) exerts its antioxidant effects in large part by generating intracellular free bilirubin via degradation of free heme; bilirubin has been shown to inhibit certain NADPH oxidase complexes in physiological intracellular concentrations [121,122,123,124,125,126]. Although the isoform specificity of this effect has not yet been adequately explored, NOX2 and NOX4 appear to be susceptible to bilirubin-mediated inhibition. A recent study reports that physiological levels of free bilirubin can inhibit activation of both inflammasomes and NF-kappaB in lipopolysaccharide (LPS)-treated macrophages [127]. Moreover, in a mouse model of LPS-induced septic shock, bilirubin injection reduces production of IL-1βand TNF-a, while enhancing survival [127].

Although bilirubin is too insoluble for oral administration, and its more soluble precursor biliverdin is an extremely expensive fine chemical, cyanobacteria (such as spirulina) as well as certain blue-green algae are very rich sources of the biliverdin derivative phycocyanobilin (PhyCB), which within cells is rapidly reduced to the bilirubin homolog phycocyanorubin; the latter appears to share bilirubin’s capacity to inhibit NADPH oxidase complexes [128,129,130]. This phenomenon likely explains the profound and versatile antioxidant and anti-inflammatory effects of orally administered spirulina (or of the spirulina protein phycocyanin, which contains PhyCB as a covalently-attached chromophore) observed in rodent models of numerous human disorders [129,131,132]. Hence, oral administration of spirulina—or of spirulina extracts enriched in PhyCB—may have clinical potential for suppressing both the priming and activation of inflammasomes. This proposition is supported by recent cell culture and rodent studies [133,134].

PhyCB might also have potential for countering the downstream effects of inflammasome activation that are mediated by IL-1β, as endosomal activation of NOX2 has been reported to play a catalytic role in IL-1β signaling [120]. Whether this might be pertinent to IL-18 is not clear.

## 4. Berberine Can Down-Regulate TXNIP Expression

Expression of TXNIP is susceptible to suppression by AMP-activated kinase (AMPK) activity. A possible explanation for this is that AMPK can interfere with the activity of a transcription factor—the carbohydrate response element-binding protein (ChREBP)—which is a key transcriptional activator of TXNIP expression. AMPK can phosphorylate this protein on serine-568; this impairs the ability of ChREBP to bind to DNA via its characteristic response elements. The contribution of ChREBP to transcription of the TXNIP gene is heightened in the context of hyperglycemia, which boosts ChREBP activity. There is also some evidence that, at least in certain contexts, AMPK can accelerate the proteasomal degradation of TXNIP. AMPK activation may also oppose inflammasome activation via activation of the deacetylase Sirt1, which has been shown to inhibit inflammasome assembly in several types of cells [135,136,137,138,139,140,141]. Sirt1 activity could be expected to inhibit inflammasome priming by suppressing the transcriptional activity of NF-kappaB, but additional mechanisms may be at play [142,143].

As is well known, the diabetes drug metformin exerts its metabolic benefits via AMPK activity. Also useful in this regard is the nutraceutical berberine, a compound found in many herbs used traditionally in Chinese medicine. Indeed, berberine is commonly employed in China for the management of type 2 diabetes. Unlike metformin, it also has the ability to lower elevated LDL cholesterol by prolonging the half-life of the mRNA coding for the hepatic LDL receptor [144,145,146]. In many cell culture and rodent models, berberine administration has been shown to suppress NLRP3 inflammasome formation [147,148,149,150,151,152].

## 5. Glucosamine May Suppress Both Priming and Activation of Inflammasomes

Pre-incubation with millimolar concentrations of glucosamine has recently been reported to suppress inflammasome activation in human and mouse macrophages primed with LPS and then activated with either ATP or nigericin (a potassium ionophore) [55]. The inhibitory effect of glucosamine on activation was associated with a failure of the inflammasome accessory proteins PKR and NEK7 to bind to NLRP3, as well as suppression of an increase in mitochondrial superoxide generation. Whether or not the impact on PKR/NEK7 was mediated by the O-GlcNAcylation of these proteins has not been determined. Glucosamine also suppressed LPS-mediated priming by impeding NF-kappaB activation. Previous studies have shown that glucosamine has the potential to oppose NF-kappaB activation via O-GlcNAcylation of the anti-inflammatory protein A20 [153]. The latter is a deubiquitinase, which reverses certain ubiquitination reactions required for TRAF6-dependent activation of NF-kappaB; its O-GlcNAcylation appears to up-regulate its activity in this regard.

Although the millimolar concentrations of glucosamine employed in these cell culture studies are markedly higher than the plasma concentrations that can be achieved in vivo with oral administration of glucosamine, it is encouraging that, when administered orally for 3 days at 250 mg/kg daily in mice, glucosamine inhibited the influx of neutrophils into the peritoneal cavity induced by subsequent intraperitoneal injection of monosodium urate crystals, which are potent stimulants of inflammasome activation [55].

## 6. Zinc Opposes Inflammasome Priming and IL-1β Generation via A20 Induction

Although zinc is known to have anti-inflammatory properties, its impact on inflammasome activity has received little direct study. However, there is reason to suspect that improvement of suboptimal zinc status may down-regulate inflammasome activation by boosting expression of the de-ubiquitinase A20 [154,155,156,157,158,159,160]. A rise in plasma zinc levels can achieve this in macrophages, monocytes, smooth muscle cells, and likely other cell types via stimulation of the G protein-coupled membrane receptor GPR39; this leads to increased transcription of the A20 gene [157,161,162].

A20 opposes inflammation by removing ubiquitination chains that serve to promote inflammatory signaling. It is best known for opposing NF-kappaB activation by removing ubiquitin chains created by the TRAF6 ubiquitinase, which form a scaffolding that functions upstream from activation of NF-kappaB and the stress-activated MAP kinases JNK and p38 [163,164,165,166]. Many agonists that promote inflammation, including those that stimulate TLR4, require TRAF6-mediated ubiquitination to exert their effects. This effect of A20 would be expected to oppose inflammasome priming that is TRAF6 dependent, and indeed there is considerable evidence that increases in A20 decrease inflammasome activity [167,168,169]. However, an additional effect is involved in this. In order to be an appropriate substrate for caspase-1, pro-IL-1β requires ubiquitination at K133 dependent on activity of the kinase RIPK3. A20 has been shown to reverse this ubiquitination, thereby reducing the ability of activated inflammasomes to generate active IL-1β [169,170].

When the mononuclear cells of healthy volunteers were stimulated ex vivo with LPS, their expression of IL1b was found to be significantly lower after the volunteers had been supplemented for 8 weeks with 45 mg zinc daily [154]. Analogously, TNF-α stimulation of NF-kappa B was found to be lower after zinc supplementation. Further study of the impact of zinc supplementation on inflammasome activity and IL-1β generation are evidently warranted. The impact of zinc supplementation on GPR activation, and hence inflammasome activity, would likely be greatest in the elderly, in whom mild zinc deficiency tends to be common [171,172,173].

Fortuitously, for reasons that remain unclear, zinc supplementation tends to boost stimulated NF-kappaB activity in lymphocytes; this effect tends to aid the activation of T lymphocytes and support cell-mediated immunity [174,175]. This likely explains why zinc supplementation can reduce the incidence of infections in the elderly [171]. Zinc thus manages to accomplish the needed trick of supporting antigen-specific immunity while curbing inflammation.

## 7. How Omega-3s Can Oppose NF-kappaB Activation

Physiological levels of the long-chain omega-3 fatty acids EPA and DHA can act as agonists for the GPR120 membrane receptor [176]. When this receptor is activated via omega-3 binding, it binds the cytoplasmic protein β-arrestin2 [177]. This interaction enables β-arrestin2 in turn to bind and sequester transforming growth factor-β activated kinase-1 (TAK1) binding protein-1 (TAB1), whose interaction with TAK1 is required for NF-kappaB activation triggered by a range of cytokines, bacterial products or DAMPs (damage-associated molecular patterns, such as high-mobility group box 1—HMGB1) that signal via TRAF2 or TRAF6; this includes toll-receptor 4 (TLR4) signaling [178]. Sequestration of TAB1 by the GPR120-β-arrestin2 complex prevents its interaction with TAK1, hence suppressing NF-kappaB activation via this type of signaling. Therefore, long-chain omega-3s have been shown to inhibit NLRP3 inflammasome activation via interaction with GPR120 [178].

Curiously, other receptors—the kappa opioid receptor, and beta2 adrenergic receptors—when activated by their agonists, have been shown to bind β-arrestin2 and thereby impede NF-kappaB activation in certain circumstances [179,180]. The activated melatonin receptors MT1 and MT2 likewise bind to β-arrestins, and it would be of interest to determine whether melatonin might reduce NF-kappaB activation by a similar mechanism [181].

## 8. Supporting Hydrogen Sulfide Synthesis

Physiological concentrations of the endogenously generated gas hydrogen sulfide (H_2_S) have been found to inhibit inflammasome activity in preclinical studies [182,183,184,185]. This may reflect, at least in part, its ability to act both as a phase 2 inducer, and as an activator of AMPK [186,187,188,189,190]. The latter effect is contingent on boosted activity of calmodulin-dependent kinase kinase-β, which confers an activating phosphorylation on AMPK [191,192,193]. Nutraceutical modulation of endogenous H_2_S production has so far received little study. Since cysteine is the rate-limiting substrate for H_2_S production via both cystathionine-beta-synthase (CBS) and cystathionine-gamma-lyase (CES), and since intracellular cysteine availability tends to decline with advancing age, supplementation with N-acetylcysteine, a nutraceutical that functions as a delivery form for cysteine, may be useful for boosting H_2_S production, particularly in the elderly [194,195]. In addition, in rodent studies, taurine supplementation has been shown amplify expression of CBS in the vascular system and of CES in the brain; the mechanistic basis of this effect remains obscure, but it is likely to account, at least in part, for the favorable impact of taurine on cardiovascular health and CNS protection observed in rodent studies [196,197].

CES is allosterically activated by S-adenosylmethionine; such activation logically would be expected to boost endogenous H_2_S expression in the brain and retina, where CES is the predominant source of H_2_S production [198,199,200]. Nutraceuticals that promote methyl group donation—folate, vitamin B12, and betaine—can be expected to help maintain an effective cellular pool of S-adenosylmethionine, and thereby promote H_2_S generation via CES.

## 9. Curcumin and Inflammasomes

Cell culture and rodent studies suggest that the commonly discussed phytochemicals curcumin and resveratrol have potential for suppression of inflammasome activation [201,202,203,204,205,206,207,208]. However, the clinical pertinence of these observations can be doubted, owing to rapid intestinal conjugation and, in the case of curcumin, poor absorption and rapid intestinal reduction in humans [209,210,211,212]. Nonetheless, efforts to develop delivery systems that might surmount these obstacles are ongoing [213,214]. Although the curcumin metabolite tetrahydrocurcumin has some anti-inflammatory properties, it does not appear to inhibit inflammasome activation [208].

## 10. Overview

These considerations suggest that nutraceutical regimens providing some or all of lipoic acid, ferulic acid, PhyCB, berberine, N-acetylcysteine, glucosamine, taurine, folate, vitamin B12, and betaine may have clinical potential for suppressing the contribution of NLRP3 inflammasomes to a number of inflammation-linked pathologies in which such inflammasomes play a key mediating role.

## 11. Pertinence to Dry Macular Degeneration

As an illustrative example, consider the dry form of age-related macular degeneration (geographic atrophy), for which there is still no clinically established therapy aside from lutein/zeaxanthin support for the macular pigment. Inflammasome-mediated production of interleukin-18 within retinal pigment epithelial cells is now strongly suspected to play a key role in driving this disorder [215,216,217,218,219,220,221,222]. The utility of supplemental zinc (80 mg daily + 2 mg copper) for slowing progression of early age-related macular degeneration (AMD) has been demonstrated in the AREDS1 trial [223,224]. Recent epidemiology has established that diabetics treated with the drug metformin are at notably lower risk for AMD than other diabetics that are comparably controlled; this could reflect a protective role for AMPK in this syndrome [225,226]. Urinary levels of the melatonin metabolite 6-sulfatoxymelatonin have been reported to be low in patients with AMD, and melatonin administration is protective in a mouse model of dry AMD [227,228,229,230]. Although the decreased risk for AMD associated with frequent spinach ingestion has been attributed to lutein, little attention has been paid to the fact that spinach is extraordinarily rich in betaine, and also a fine source of folate. In the first epidemiological study associating lutein ingestion with AMD risk, Seddon and colleagues reported that subjects who claimed to consume spinach or collard greens almost every day were at 80% lower risk for AMD [231]. Whether this magnitude of protection could be confirmed in further studies remains to be seen, but this might be an intriguing hint that the betaine content of spinach also contributes to the protection it affords. Indeed, a study in identical twins discordant for AMD found that diets high in betaine were associated with protection from this disorder [232]. Moreover, in a placebo-controlled clinical trial, which so far has received insufficient attention, supplementation with folic acid (2.5 mg) and vitamins B12 and B6 was associated with a reduction in risk for new-onset AMD of about one-third after 7 years of follow-up [233]. Conversely, elevated homocysteine, a marker for poor methyl group availability, is an established risk factor for AMD [234]. Conceivably, these findings reflect a modulatory effect of methyl donors on CES activity in the retina, though the possibility that modulation of DNA methylation plays some role in this phenomenon might be considered.

## 12. Pertinence to the Management of COVID-19

It is notable that PhyCB, phase 2 inducers, melatonin, N-acetylcysteine, berberine, glucosamine, and zinc have already been suggested for use in the management of COVID-19 [235,236,237,238,239,240,241,242,243,244,245]. The NADPH oxidase-inhibitory activity PhyCB may have potential for up-regulating the type 1 interferon response to SARS-CoV-2 and other RNA viruses, and may also act to suppress the induction of tissue factor, a likely trigger for the thrombotic complications of COVID-19 [235,236]. Phase 2 inducers and N-acetylcysteine could be expected to play a complementary role with respect to these effects [235,236]. Glucosamine may likewise up-regulate the type 1 interferon response to viruses [236,246]. Whereas the impact of berberine on COVID-19 has not yet been assessed, use of metformin—like berberine, an activator of AMPK—has been associated with lower risk for mortality in diabetic patients hospitalized with COVID-19 [247,248]. A lower blood zinc level has been correlated with a poorer clinical outcome in early-stage COVID-19 patients treated with hydroxychloroquine and azithromycin [249]. However, whether short-term zinc supplementation can importantly influence zinc status in COVID-19 patients is questionable, and the utility of supplemental zinc in this context is the subject of conflicting reports [250,251,252]. Long-term supplementation prior to infection may be the best protective strategy. The possibility that treatment with zinc ionophores (such as chloroquine) might enable zinc to act intracellularly to inhibit replications of coronaviruses has been suggested; while this phenomenon has been demonstrated in vitro, its relevance in vivo remains to be established [253,254]. A fall in serum levels of H_2_S has been associated with increased mortality in hospitalized COVID-19 patients, whereas these serum levels correlate inversely with the inflammation markers interleukin-6 (IL-6) and C-reactive protein, known markers for an adverse outcome; hence, nutraceuticals that support H_2_S production may have a protective anti-inflammatory impact in this syndrome [255]. It is of possible relevance that taurine administration has been shown to exert anti-inflammatory activity when the lungs of rodents have been challenged with endotoxin or various other pro-inflammatory agents [256,257,258]. Similar effects have been reported with phycocyanin, glucosamine, berberine, lipoic and ferulic acids, melatonin, and N-acetylcysteine [259,260,261,262,263,264,265].

Clinical studies suggest that the drug colchicine may be useful in the cytokine storm phase of COVID-19 [266,267,268]. It is therefore intriguing to consider the fact that colchicine can suppress activation of NLRP3-dependent inflammasome activation by disrupting microtubules required for achieving the apposition of mitochondrially-bound ASC with endoplasmic reticulum-bound NLRP3 necessary for inflammasome formation [269,270]. Colchicine’s activity in gout may be partially rooted in this anti-inflammasome mechanism, as IL-1β is a key mediator of gouty arthritis [29]. Unfortunately, colchicine’s narrow therapeutic index lessens the feasibility of its use in preventive medicine [271].

The lethality of COVID-19 is notably greater in patients with metabolic syndrome: visceral obesity, hypertension, diabetes [272,273,274,275]. Arguably, this may reflect greater exposure of lung tissue to saturated fatty acids such as palmitic acid, that can exert pro-inflammatory effects [276]. One such effect of palmitic acid is an up-regulation of inflammasome activation [277,278,279]. Hence, nutraceutical measures that oppose inflammasome activation may offset to some degree the greater mortality risk imposed by metabolic syndrome in COVID-19 patients.

## 13. Summation

**Figure 1:**
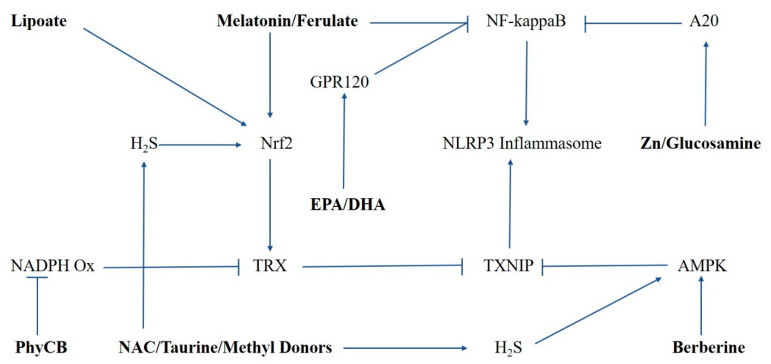
Mechanisms for nutraceutical suppression of NLRP3 inflammasome activation. TRX, thioredoxin; TXNIP, thioredoxin interacting protein.

NLRP3-dependent inflammasomes play a mediating role in a vast range of inflammation-linked pathologies, by catalyzing the formation and extracellular export of interleukins-1β and -18, and by inducing pyroptotic cell death. A consideration of the molecular biology underlying inflammasome priming and activation, and of pertinent scientific literature, suggests that a number of nutraceuticals may have potential for blunting inflammasome activity. A range of antioxidant measures may oppose the oxidant-catalyzed association of TXNIP with NLRP3, including PhyCB, phase 2 inducers (e.g., lipoic acid, ferulic acid, melatonin), and N-acetylcysteine. AMPK activators such as berberine oppose the expression of TXNIP at the transcriptional level. Glucosamine can work to oppose the NF-kappaB activation required for inflammasome priming, and also by inhibiting the association of NLRP3 with the accessory proteins PKR and NEK7, likely by promoting O-GlcNAcylation of key proteins. Zinc can oppose inflammasome priming and IL-1β generation via induction of the A20 de-ubiquitinase. Since H_2_S inhibits inflammasome activity, nutraceuticals that support H_2_S synthesis—N-acetylcysteine, and, in at least certain circumstances, taurine and catalysts of methyl donation—may aid this effect. These relationships are summarized in the Figure 1. Complex nutraceutical programs or functional foods featuring these agents may have preventive and therapeutic utility in the diverse range of pathologies in which inflammasome activity plays a mediating role.

As a final comment, it has recently been reported that NLRP3-knockout mice enjoy a considerable enhancement of both mean (34%) and maximal (29%) lifespan [280]. This makes the possibility of safe nutraceutical and dietary measures for down-regulating inflammasome activity all the more intriguing.

Since the writing of the original draft of this manuscript, a comprehensive review has appeared citing literature on a vast range of phytochemicals that have modulated inflammasome activity in vitro or in vivo [281]. This review may be considered complementary to ours. Our focus is on nutraceuticals that are currently commercially available, can be presumed to be adequately absorbed and physiologically active in defined oral dose schedules, and have reasonably well-defined mechanisms of action. Hence, it lends itself to current development of functional foods and supplementation programs, which may have some clinical utility for controlling inflammasome activation.

## Data Availability

Data sharing not applicable.
